# Effects of Whole Brown Bean and Its Isolated Fiber Fraction on Plasma Lipid Profile, Atherosclerosis, Gut Microbiota, and Microbiota-Dependent Metabolites in *Apoe*^−/−^ Mice

**DOI:** 10.3390/nu14050937

**Published:** 2022-02-22

**Authors:** Jiyun Liu, Mohammed E. Hefni, Cornelia M. Witthöft, Maria Bergström, Stephen Burleigh, Margareta Nyman, Frida Hållenius

**Affiliations:** 1 Department of Chemistry and Biomedical Sciences, Faculty of Health and Life Sciences, Linnaeus University, 39231 Kalmar, Sweden; mohammed.hefni@lnu.se (M.E.H.); cornelia.witthoft@lnu.se (C.M.W.); maria.bergstrom@lnu.se (M.B.); 2 Food Industries Department, Faculty of Agriculture, Mansoura University, Mansoura 35516, Egypt; 3 Department of Food Technology, Engineering and Nutrition, Lund University, 22100 Lund, Sweden; stephen.burleigh@food.lth.se (S.B.); margareta.nyman@food.lth.se (M.N.); frida.hallenius@food.lth.se (F.H.)

**Keywords:** brown bean, dietary fiber, atherosclerotic plaques, short-chain fatty acid, trimethylamine *N*-oxide, gut microbiota, *Apoe*^−/−^ mice

## Abstract

The health benefits of bean consumption are widely recognized and are largely attributed to the dietary fiber content. This study investigated and compared the effects of whole brown beans and an isolated bean dietary fiber fraction on the plasma lipid profile, atherosclerotic plaque amount, gut microbiota, and microbiota-dependent metabolites (cecal short-chain fatty acids (SCFAs) and plasma methylamines) in *Apoe*^−/−^ mice fed high fat diets for 10.5 weeks. The results showed that both whole bean and the isolated fiber fraction had a tendency to lower atherosclerotic plaque amount, but not plasma lipid concentration. The whole bean diet led to a significantly higher diversity of gut microbiota compared with the high fat diet. Both bean diets resulted in a lower Firmicutes/Bacteroidetes ratio, higher relative abundance of unclassified *S24-7*, *Prevotella*, *Bifidobacterium*, and unclassified *Clostridiales*, and lower abundance of *Lactobacillus*. Both bean diets resulted in higher formation of all cecal SCFAs (higher proportion of propionic acid and lower proportion of acetic acid) and higher plasma trimethylamine *N*-oxide concentrations compared with the high fat diet. Whole beans and the isolated fiber fraction exerted similar positive effects on atherosclerotic plaque amount, gut microbiota, and cecal SCFAs in *Apoe*^−/−^ mice compared with the control diets.

## 1. Introduction

Pulse foods are attracting attention due to their health benefits, especially in the prevention of metabolic and cardiovascular diseases [1,2,3]. For example, a cross-sectional study of Iranian adults showed that pulse consumption decreased the risk of metabolic diseases [1], and an epidemiological follow-up study indicated that pulse intake is associated with a decreased risk of heart disease [2]. A meta-analysis of randomized controlled trials showed that a diet rich in pulses can decrease total and low-density lipoprotein (LDL) cholesterol in blood [3].

The beneficial effects of pulses are often associated with their high content of dietary fiber (non-starch polysaccharides) and other indigestible carbohydrates (resistant starch and oligosaccharides) and polyphenols [4,5]. Among all of the pulse types, common beans (*Phaseolus vulgaris* L.) contain the highest level of fermentable fiber and have the highest in vitro fermentability [6]. 

Gut microbiota also play an important role in maintaining metabolic health. Dysbiosis, characterized by a decrease in microbial diversity and an increase in proinflammatory species, has been linked with obesity, diabetes, and atherosclerotic cardiovascular disease [7,8]. Lower abundance of Bacteroidetes and proportionally higher abundance of Firmicutes has been found in obese subjects compared with lean subjects in both human and mouse studies [9,10]. Diet plays an important role in shaping the gut microbiota [11,12]. Positive effects of dietary pulses on gut microbiota have been reported in both in vitro and in vivo studies [13,14].

The effects of gut microbiota are often mediated by their metabolites. Short-chain fatty acids (SCFA) have an important role in health and diseases, as they are the main metabolites produced by gut microbiota from indigestible carbohydrate [15]. The SCFA profile depends on several factors, such as the physicochemical properties and monomeric composition of the fiber and the composition of the gut microbiota [16]. SCFAs show activity in maintaining colonic health, integrity, and immunity, with butyric acid being the most potent member, followed by propionic and acetic acid [15]. Most of the known butyric acid-producing bacteria belong to the phylum Firmicutes [17].

Trimethylamine *N*-oxide (TMAO) is another important microbiota-dependent metabolite. It can be produced from trimethylamine (TMA) in the liver, while TMA is generated by gut microbiota from precursors such as choline and carnitine [18]. Various bacterial phyla, including Firmicutes, Proteobacteria, and Actinobacteria, are capable of producing TMAO [19]. The increased concentration of TMAO in plasma resulting from gut microbiota dysbiosis is suggested to be a risk factor for atherosclerosis [20].

Brown bean (*Phaseolus vulgaris* L.) is one of the most important types of pulses consumed in Sweden. A previous study showed that an evening meal of brown beans can beneficially alter blood glucose, insulin, and appetite regulatory hormones, and promote plasma SCFA concentrations in human subjects [21]. However, the effects of brown beans on plasma lipid profile, regulation of gut microbiota, and prevention of atherosclerosis have not been fully studied. The role of dietary fiber of beans has also not been fully investigated. The aim of this study was therefore to evaluate and compare the effects of whole brown beans and the isolated dietary fiber fraction on lipid profile, atherosclerotic plaque amount, gut microbiota, and microbiota-dependent metabolites in *Apoe*^−/−^ mice fed a high fat (HF) diet.

## 2. Materials and Methods

### 2.1. Preparation of Whole Bean and Fiber Fraction

Brown bean (*Phaseolus vulgaris* L. var. *Katja*) obtained from Kalmar-Ölands Trädgårdsprodukter (KÖTP, Färjestaden, Sweden) was used for the preparation of lyophilized boiled whole brown bean (Wbean) and a brown bean fiber (Bfiber) fraction to be incorporated into experimental diets. The beans were prepared following the cooking instructions provided by KÖTP. In brief, after overnight soaking (14 h) and boiling in water for 60 min, the cooked brown beans were collected and lyophilized (BenchTop Pro, SP VirTis, Gardiner, NY, USA), milled (Cyclotec 1093, Foss Tecator, Hillerød, Denmark), and stored at −20 °C.

The preparation of the Bfiber fraction from lyophilized boiled Wbean followed the protocol provided by Megazyme Ltd. (Bray, Ireland) (based on AOAC Method 985.29), with some modifications. In brief, the bean lyophilizate was mixed with distilled water to a final volume of 100 g/L. Thermostable α-amylase was added and the solution was incubated in a boiling water bath for 45 min. After cooling, protease was added to hydrolyze the proteins at 60 °C [22] for 45 min. Then, 0.5 M HCl was used to adjust the pH of the suspension to 4.8 and amyloglucosidase was added, followed by incubation at 60 °C [23] for another 45 min. Four times volume of ethanol (95%) was added to the dispersion to precipitate the fiber. The Bfiber fraction was filtered and then washed with 80% ethanol. The residue was lyophilized to obtain the bean fiber fraction.

The Wbean and the Bfiber fraction were shipped to Research Diets (New Brunswick, NJ, USA) for preparation of the experimental diets. There were two control diets (a high fat (HF) and a low fat (LF) diet) and two bean (Wbean and Bfiber) high fat diets. Each diet included 60 g/kg of cellulose or dietary fiber from beans. Corn starch was used to adjust dry matter content. A detailed description of the diets is given in Table S1 in the Supplementary Material.

### 2.2. Design of Animal Study

Six-week-old female *Apoe*^−/−^ mice obtained from Taconic Biosciences Inc. (Silkeborg, Denmark) were randomly divided into four groups, with 10 mice per group. They were housed five per cage in a controlled environment (12 h day/night cycle, 22 °C) with feed and water ad libitum. At the beginning of the study, the mice were allowed to adapt to the environment for one week with regular chow pellets. The diet was then changed to one of the four experimental diets (HF, LF, Wbean, and Bfiber) for each group (Table S1 in the Supplementary Material). During the 10.5-week feeding period, the weight of the mice and the diet consumption in each cage were recorded weekly. One mouse in the LF group and one in the Wbean group had skin irritation during the feeding period (related to the poor physical constitution of individual mice, rather than the diets), and were excluded from the study. At the end of the feeding period, the mice were anesthetized in chambers saturated with isoflurane and then sacrificed by cardiac puncture. Blood (1 mL) was collected and immediately centrifuged to separate the plasma. The liver and fat pads were weighed. The heart, including the aortic arch, was harvested. The cecal tissue and contents were weighed, collected, and stored at −80 °C for analysis of SCFAs and gut microbiota. 

The animal study was approved by the Malmö–Lund Ethics Committee on Animal Experiments (ethical approval number: 9874-20).

### 2.3. Analytical Methods

#### 2.3.1. Characterization of Whole Bean and the Fiber Fraction

*Dietary fiber.* The content of dietary fiber in Wbean and the Bfiber fraction was measured using a commercially available total dietary fiber kit (Megazyme, Ltd., Bray, Ireland). The neutral sugar composition of the fiber was determined using GC–FID and the content of uronic acids was determined colorimetrically [24].

*Protein, fat, ash, and carbohydrate.* The content of the total protein was determined according to AOAC method 992.15, fat content according to AOAC method 989.05, moisture content according to AOAC method 925.10, and ash content according to AOAC method 923.03. Carbohydrate content was determined by difference (i.e., 100%—%(Fat + Protein + Fiber + Moisture + Ash)). 

#### 2.3.2. Analysis of Biological Samples

*Lipid profiles in plasma.* Plasma levels of triglycerides (TG), total cholesterol, high-density lipoprotein (HDL) cholesterol, and LDL cholesterol were determined using colorimetric kits (Thermo Fisher Scientific Inc., Waltham, MA, USA).

*Atherosclerotic plaques.* Mice hearts (2–5 mice per group) were embedded in OCT cryomount medium and sent to Histocenter (Gothenburg, Sweden) for the preparation of slides. The hearts were cut into 10 μm sections for histological analysis of atherosclerotic plaques. After staining with Oil Red O (ORO) (Histolab, Gothenburg, Sweden) and hematoxylin (MAYERS HTX, Histolab, Gothenburg, Sweden), representative images of aortic sections were obtained using a camera–microscope system (Nikon Eclipse E400) with imaging software (NIS-Elements 4.13) (both Nikon Instruments Inc., Tokyo, Japan). The atherosclerotic plaque amount (ratio of plaque area to total vessel area, %) was quantified using BioPixQ 2.0 software (BIOPIX software, Gothenburg, Sweden).

*Creatinine and methylamines in plasma*. The creatinine and methylamines in plasma were measured as previously described with some modifications [25]. In brief, the plasma samples (25 μL) were mixed with internal standard (50 μmol/L, 10 μL, containing betaine-d11, trimethylamine-d9, trimethylamine-N-oxide-d9, creatinine-d3, L-carnitine-d3, acetyl-DL-carnitine-d3), iodoacetonitrile (5 μL), NH_4_OH (2 μL), and CH_3_CN: CH_3_OH (90:10, *v*/*v*, 458 μL) and vortexed for 5 min, centrifuged (13,000× *g*, 5 min), and filtered through a 0.45 μm Millipore filter (Agilent, St. Clara, CA, USA). The supernatant was collected for LC–MS analysis (Agilent 1200, Agilent Technologies, Santa Clara, CA, USA). Methylamines and creatinine were separated using a neutral HILIC column (ACE, 150 mm × 4.6 mm; particle size 3 μm). The mobile phase consisted of 25 mM ammonium formate in water: CH_3_CN (30:70, *v*/*v*). The flow rate was set to 0.6 mL/min and the injection volume to 10 μL. Quantification was based on external calibration and internal standards. 

*SCFAs in cecum.* The content and profile of SCFAs (acetic, propionic, butyric, isobutyric, valeric, isovaleric, caproic, and heptanoic acids) in the cecum were analyzed using a GC–FID method [26] with 2-ethylbutyric acid as the internal standard. In brief, hydrochloric acid (0.25 M) was mixed with the samples and vortexed for 5 min. The suspension was then centrifuged (8000× *g*, 20 min), and the supernatant was collected and injected into an Agilent DB-FFAP column (30 m × 0.53 mm, 0.50 μm film thickness) for GC analysis (Agilent GC7890B). The parameters were set as: splitless mode, flow of carrier gas 4.4 mL/min, and temperature of injection port 250 °C. The temperature gradient was as follows: 0–2 min, 80 °C; 2–14.5 min, 80–180 °C; 14.5–15.5 min, 180 °C; 15.5–16.5 min, 180–200 °C; 16.5–21.5 min, 200 °C.

### 2.4. 16S rRNA Gene-Based Gut Microbial Analysis

Bacterial genomic 16S rRNA gene sequencing was carried out by Clinical Microbiomics Laboratory (Copenhagen, Denmark). In brief, bacterial DNA was extracted from cecal content by bead-beating, using the QIAamp DNA Stool Mini kit (Qiagen, Limburg, Germany). The 27F forward (AGAGTTTGATCCTGGCTCAG) and 534R reverse (ATTACCGCGGCTGCTGG) primers containing adapter sequences (Illumina, San Diego, CA, USA) with unique dual indices to tag each PCR product were used to amplify genes by PCR [27]. Paired-end sequencing with a read length of 2 × 300 bp was performed on a Miseq instrument (Illumina), using a Miseq Reagent kit v3 (Illumina). The 16S rRNA gene sequences were analyzed using Quantitative Insights into Microbial Ecology (QIIME, version 1) for high-throughput community data. After quality filtering, a total of 1,181,458 sequences of operational taxonomic units (OTUs) were generated. An average count of 31,931 sequences was assigned to each sample (range 18,108 to 56,608 sequences/sample). The sequences were normalized by rarefaction (depth of 18,000), grouped into OTUs at a minimum of 97% similarity using the closed reference method based on the Greengenes database (v. 13.8), and filtered by the removal of singletons and low abundance OTUs (minimum count fraction set at 0.001). 

### 2.5. Statistical Analyses 

GraphPad Prism 9.2.0 (GraphPad, La Jolla, CA, USA) and Excel (Microsoft, Redmond, WA, USA) were used for univariate analysis. Unless otherwise stated, the differences between groups were assessed by Student’s *t*-test or by one-way ANOVA, followed by Tukey’s multiple comparison test. Welch’s ANOVA test followed by Dunnett’s T3 multiple comparison test was used when the requirement of equal standard deviations (SDs) was not fulfilled. For the non-parametric taxonomy data, Wilcox test followed by Dunn’s test for multiple comparisons was applied. The significance was set to *p* < 0.05, while *p* < 0.1 was considered to indicate a tendency. 

For multivariate analysis, a QIIME-based permanova (using the pseudo-F statistical test and 999 permutations) was used to test for overall differences in the microbiomes between groups. Spearman’s correlation analysis was performed using Prism 9.2.0 (GraphPad, La Jolla, CA, USA) with *p* < 0.05 to explore the correlations between TMAO, gut microbiota, and atherosclerotic plaque amount. Principal component analysis (PCA) was performed using MATLAB (MathWorks, Natick, MA, USA) to explore the correlations of different diets to the biomarkers and gut microbiota. 

## 3. Results

### 3.1. Characterization of Whole Bean and the Fiber Fraction

The total content of dietary fiber in whole brown beans and in the fiber fraction isolated from brown beans was 27 and 56 g/100 g dry weight (dwt), respectively. The fiber consisted mainly of arabinose (36%), followed by glucose (29%) and uronic acids (13%). Considerable amounts of xylose (10%), galactose (7%) and fucose (3%) were also found (Figure S1 in the Supplementary Material). Polyphenolic compounds (513 μg/g) were detected only in the whole bean samples (Table S2 in the Supplementary Material).

### 3.2. Diet Intake, Weight Gain, and Organ Weight Ratios

Mice in the LF group showed 19% lower cecum-to-body weight ratio compared with mice in the HF group (*p* < 0.05). No significant differences in body weight gain or other organ weight ratios were observed between the controls. 

Mice fed the two bean diets had significantly higher diet intake and body weight gain compared with mice in the HF group (*p* < 0.0001). The Bfiber group had a higher cecum-to-body weight ratio compared with the HF group (*p* < 0.05) (Figure S2 in the Supplementary Material).

### 3.3. Plasma Lipid Profiles and Atherosclerotic Plaque 

The LF group had lower total and LDL cholesterol levels (*p* < 0.0001), and lower atherosclerotic plaque amount (*p* < 0.001) compared with the HF group. 

**Figure 1:**
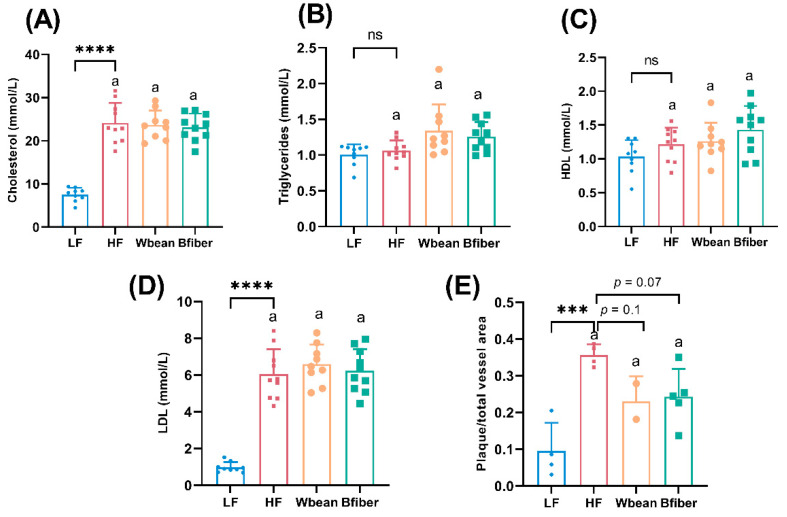
Plasma levels of (**A**) total cholesterol, (**B**) total triglycerides, (**C**) high density lipoprotein (HDL) cholesterol, (**D**) low density lipoprotein (LDL) cholesterol, and (**E**) atherosclerotic plaque amount in the aortic root of mice fed different diets for 10.5 weeks. Values in low fat (LF) and high fat (HF) groups were compared using Student’s *t*-test (or Welch’s *t*-test for data with unequal SDs). Values in the high fat groups, i.e., HF, whole brown bean (Wbean), and bean fiber (Bfiber), were compared using one-way ANOVA (Welch’s ANOVA test for data with unequal SDs, or Kruskal–Wallis test for nonparametric data) followed by post hoc tests. Different lowercase letters within panels indicate significant differences (*p* < 0.05). ns: not significant. *** *p* < 0.001, **** *p* < 0.0001.

No significant differences in the plasma lipid profile were observed between the HF and two bean groups. Inclusion of Wbean or Bfiber in the HF diet resulted in a tendency (*p* = 0.1 and 0.07, respectively) for lower atherosclerotic plaque amount compared with the HF diet (Figure 1).

### 3.4. Cecal SCFAs

The LF diet resulted in 8% lower proportion of acetic acid (*p* < 0.01), and 30% higher pool and 25% higher proportion of butyric acid in cecum, compared with the HF diet (*p* < 0.05).

**Figure 2:**
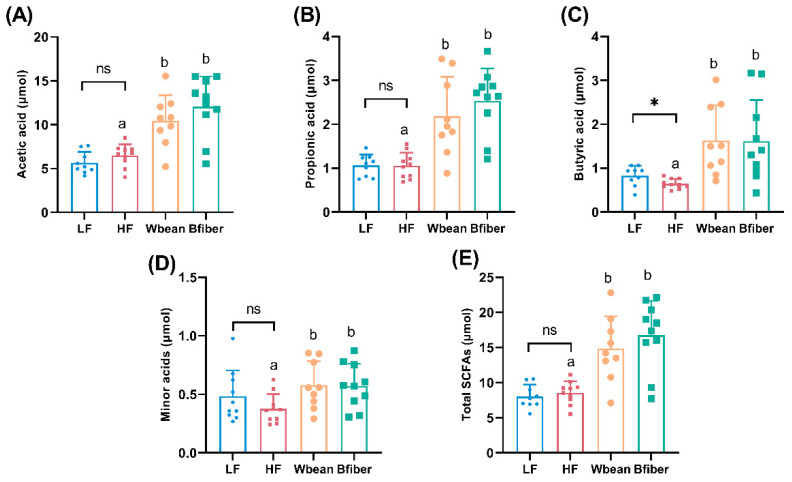
Cecal pools (μmol) of (**A**) acetic acid, (**B**) propionic acid, (**C**) butyric acid, (**D**) minor acids and (**E**) the total short-chain fatty acids (SCFAs) in *Apoe*^−/−^ mice fed a control diet (low fat (LF) or high fat (HF)) or a bean diet (whole brown bean (Wbean), bean fiber (Bfiber)) in a HF setting for 10.5 weeks. Minor acids include isobutyric acid, valeric acid, isovaleric acid, caproic acid, and heptanoic acid. Values in the LF and HF controls were compared using Student’s *t*-test. Values in the high fat groups, i.e., HF, Wbean, and Bfiber, were compared using one-way ANOVA followed by Tukey’s multiple comparison test. Different lowercase letters and symbol (*) within panels indicate significant differences (*p* < 0.05). ns: not significant.

Mice fed the Wbean or Bfiber diet had higher cecal pools of total and individual SCFAs (*p* < 0.05) than mice fed the HF diet. Both bean diets also led to lower proportions of acetic acid and higher proportions of propionic acid compared with the HF diet. Mice fed the Wbean diet showed a tendency (*p* = 0.1) for a higher proportion of butyric acid compared with mice fed the HF diet. Besides, mice fed the Bfiber diet had a lower proportion (*p* < 0.05) of minor acids (including isobutyric, valeric, isovaleric, caproic, and heptanoic acids) compared with mice fed the HF diet. There were no significant differences between the two bean groups (Figure 2 and Figure S3 in the Supplementary Material). 

### 3.5. Plasma Methylamines

The LF diet led to higher plasma concentrations of TMAO (*p* < 0.05) and L-carnitine (*p* < 0.01), and lower concentrations of creatinine (*p* < 0.05), compared with the HF diet.

**Figure 3:**
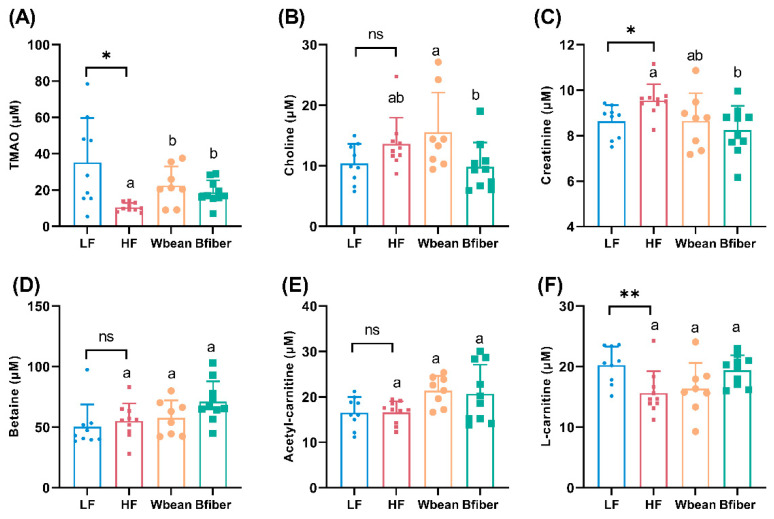
Concentrations (μM) of plasma (**A**) trimethylamine *N*-oxide (TMAO), (**B**) choline, (**C**) creatinine, (**D**) betaine, (**E**) acetyl-carnitine, and (**F**) L-carnitine in *Apoe*^−/−^ mice fed the low fat (LF), high fat (HF), whole brown bean (Wbean), or bean fiber (Bfiber) diet for 10.5 weeks. Trimethylamine (TMA) concentrations were low (<3 μM) in all groups and no significant differences were observed. Values in the LF and HF controls were compared using Student’s *t*-test. Values in the high fat groups, i.e., HF, Wbean, and Bfiber, were compared using one-way ANOVA (Welch’s ANOVA test for data with unequal SDs, or Kruskal–Wallis test for nonparametric data) followed by post hoc tests. Different lowercase letters within panels indicate significant differences (*p* < 0.05). ns: not significant. * *p* < 0.05, ** *p* < 0.01.

Mice fed the Wbean or Bfiber diets had around twice the plasma TMAO concentrations (*p* < 0.05) seen in mice fed the HF diet. The Bfiber diet resulted in lower plasma creatinine concentrations (*p* < 0.05) compared with the HF diet, while the Wbean diet gave a lowering tendency (*p* = 0.09). The plasma choline concentration was higher in the Wbean group than the Bfiber group (*p* < 0.05) (Figure 3). 

### 3.6. Diversity of Gut Microbiota

**Figure 4:**
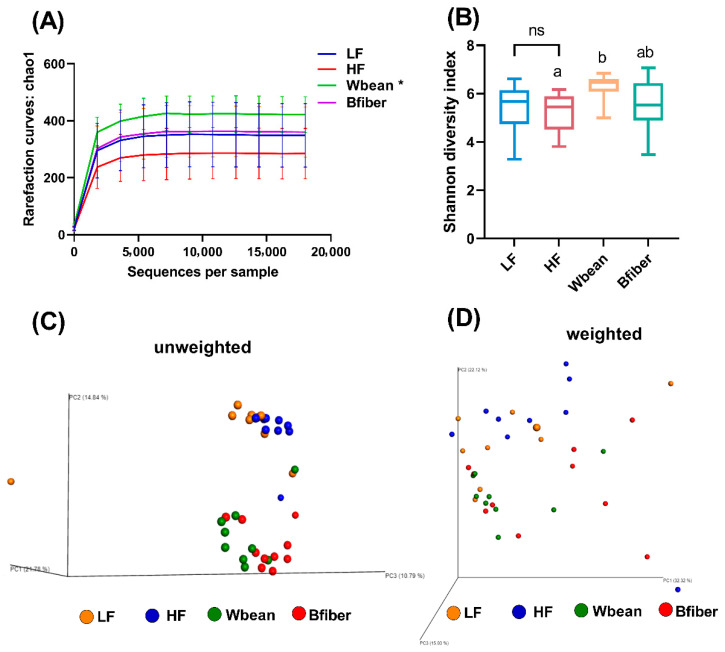
(**A**) Chao1 rarefaction curves, (**B**) Shannon diversity index, and (**C**) unweighted and (**D**) weighted UniFrac principal coordinates analysis (PCoA) plots obtained from analyses of cecal samples from mice fed the low fat (LF), high fat (HF), whole brown bean (Wbean), or bean fiber (Bfiber) diet for 10.5 weeks. Chao1 index values were compared using Student’s *t*-test for the two control groups and using Kruskal–Wallis test followed by Dunn’s multiple comparison test for the high fat groups, i.e., HF, Wbean, and Bfiber. Different lowercase letters and symbol (*) within panels indicate significant differences (*p* < 0.05). ns: not significant.

Alpha diversity was measured using Chao 1 estimator from rarefaction curves (Figure 4A) and Shannon diversity index (Figure 4B). The rarefaction curves at a range of sampling depths suggested that 31,931 sequences per sample was sufficient. The Wbean group had higher alpha diversity compared with the HF control, according to both the Chao 1 estimator (*p* < 0.05) and Shannon diversity index (*p* < 0.05) (Figure 4A,B).

Beta diversity was illustrated by principal coordinates analysis (PCoA) plots of unweighted and weighted UniFrac matrices (Figure 4C,D). Clear separation of cecal microbiota between the mice fed the control diets and the mice fed the bean intervention diets was observed. Moreover, the distances between the control and bean intervention groups were larger in the unweighted UniFrac matrix than in the weighted matrix, suggesting that the groups shared more similarity in the taxa with high relative abundance than those with low abundance. 

### 3.7. Relative Abundance of Gut Microbiota

**Figure 5:**
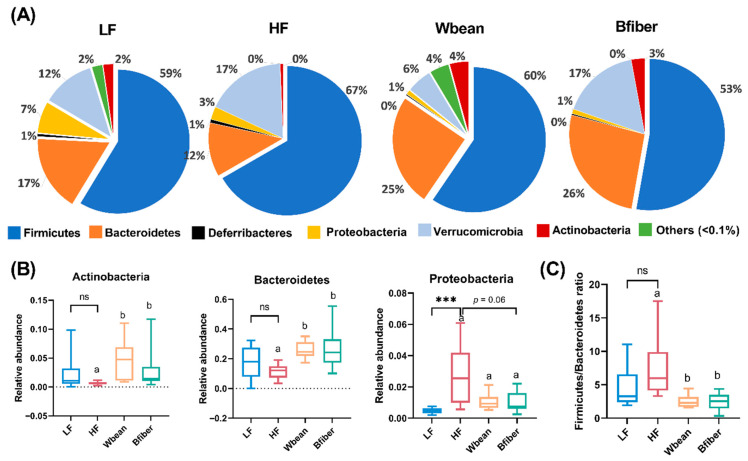
(**A**) Relative abundance at phylum level of different gut microbial taxa and significant differences in (**B**) phyla and (**C**) Firmicutes/Bacteroidetes ratio detected for cecal samples from mice fed the low fat (LF), high fat (HF), whole brown bean (Wbean), or bean fiber (Bfiber) diet for 10.5 weeks. Values in the LF and HF groups were compared using Welch’s *t*-test (or Mann–Whitney test for nonparametric data). Values in the high fat groups, i.e., HF, Wbean, and Bfiber, were compared using one-way ANOVA (Welch’s ANOVA test for data with unequal SDs, or Kruskal–Wallis test for nonparametric data) followed by post hoc tests. Different lowercase letters within panels indicate significant differences (*p* < 0.05). ns: not significant. *** *p* < 0.001.

**Figure 6:**
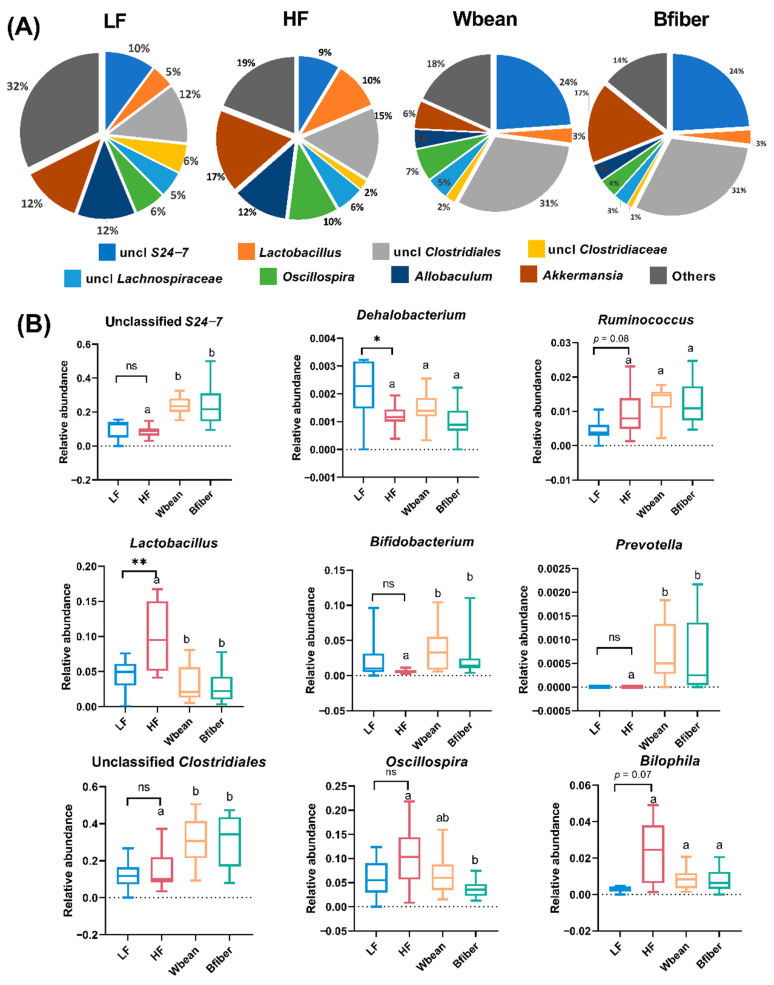
(**A**) Microbial profile at genus level and (**B**) relative abundance of genera showing a significant difference or tendency in the cecum of mice fed a low fat (LF), high fat (HF), whole brown bean (Wbean), or bean fiber (Bfiber) diet for 10.5 weeks. Values in the LF and HF groups were compared using Welch’s *t*-test (or Mann–Whitney test for nonparametric data). Values in the high fat groups, i.e., HF, Wbean, and Bfiber, were compared using one-way ANOVA (Welch’s ANOVA test for data with unequal SDs, or Kruskal–Wallis test for nonparametric data), followed by post hoc tests. Different lowercase letters above data for the HF setting groups indicate significant differences (*p* < 0.05). ns: not significant. * *p* < 0.05, ** *p* < 0.01.

Significant differences were observed in the composition of gut microbiota between the five treatments based on a QIIME permanova (*p* < 0.01). These differences were also visually apparent at the phylum level (Figure 5A) and genus level (Figure 6A), and in the PCA biplots (Figure S4 in the Supplementary Material).

The LF diet led to lower relative abundance of Proteobacteria compared with the HF diet (*p* < 0.001). Both bean groups had higher abundances of Actinobacteria and Bacteroidetes compared with the HF group (*p* < 0.05). The Bfiber diet resulted in a tendency for lower abundance of Proteobacteria (*p* = 0.06) compared with the HF diet. Furthermore, the Firmicutes/Bacteroidetes (F/B) ratio was lower in the Wbean (*p* < 0.01) and Bfiber (*p* < 0.05) groups than in the HF group (Figure 5). 

At the genus level, unclassified *Clostridiales* (31%) and unclassified *S24-7* (24%) were the predominant species in mice fed the bean diets, while *Akkermansia* (12–17%) and unclassified *Clostridiales* (12–15%) constituted the majority in mice fed the control diets (Figure 6A). Significant differences (*p* < 0.05) or tendencies (*p* < 0.1) for differences in nine genera, i.e., unclassified *S24-7*, *Dehalobacterium*, *Ruminococcus*, *Lactobacillus*, *Bifidobacterium*, *Prevotella*, unclassified *Clostridiales*, *Oscillospira*, and *Bilophila*, were observed between groups (Figure 6B).

### 3.8. Overall Effects of Diets on Biomarkers and Gut Microbiota

The PCA biplots of the data on organ and body weight, cecal SCFAs, plasma lipids, methylamines, and gut microbiota (Figure S4 in the Supplementary Material) in the mice revealed clear differences between the bean diets and the two control groups. In both biplots, the Wbean or Bfiber groups tended to cluster together, showing similar effects of the two bean diets on biomarkers and gut microbiota. In terms of physiological parameters, the two bean intervention groups were positively correlated with parameters such as cecum weight and the amount of acetic, propionic, and total SCFAs. In terms of gut microbiota, the two bean groups were positively associated with *Clostridiales*, *Ruminococcus*, *Prevotella*, *Roseburia*, *Ruminococcaceae*, and unclassified *S24-7*.

### 3.9. Correlation between TMAO, Atherosclerotic Plaque, and Gut Microbiota

Plasma TMAO concentration was not found to be correlated with atherosclerotic plaque amount (data not shown), but it was correlated with the bacterial taxa *Mucispirillum*, *SMB53*, *Peptococcaceae*, *Anaerotruncus*, *Oscillospira*, *Sutterella*, and *Bilophila*. Atherosclerotic plaque amount was correlated to *Streptococcus* and *Ruminococcaceae* (*p* < 0.05). 

## 4. Discussion

‘Dietary fiber’ in this study refers to non-starch polysaccharides, excluding oligosaccharides and resistant starch. Beans are a good source of dietary fiber, and also contain a large amount of protein (19%) (Table S2) and some other indigestible carbohydrates (i.e., resistant starch and oligosaccharides) (up to 10 g/100 g dwt in total [21,28,29]). We evaluated and compared the in vivo effects of whole brown bean and its fiber fraction on the plasma lipid profiles, atherosclerotic plaque amount, gut microbiota, and microbiota-dependent metabolites (cecal SCFAs and plasma methylamines) in *Apoe*^−/−^ mice. Both the Wbean and Bfiber diets showed a tendency for lower atherosclerotic plaque amount compared with the HF diet, although neither diet resulted in lower body weight or lower plasma lipid concentration. The Wbean diet led to higher alpha diversity of gut microbiota. Both bean diets exhibited similar effects in the modulation of all parameters investigated. 

### 4.1. Effects of Bean Diets on Gut Microbiota

Low diversity of gut microbiota often implies gut dysbiosis and is associated with an unhealthy status of the host [30]. Alpha diversity shows the richness and evenness of microbial species in a sample, while beta diversity shows the variation in microbial communities between samples [31]. We found that the Wbean diet resulted in a higher alpha diversity of gut microbiota compared with the HF diet, while the Bfiber diet did not. Thus, the increase in alpha diversity caused by the Wbean diet may be attributable to components other than the non-starch polysaccharides in cooked beans, such as polyphenols or oligosaccharides. Similarly, diet supplementation with navy bean and black bean has been found to enhance the alpha diversity of gut microbiota in C57BL/6 mice [4]. A kidney bean hull diet was also reported to promote the alpha diversity in rats [32]. There was a clear clustering of the Wbean and Bfiber groups in the unweighted UniFrac PCoA plot (Figure 4C) and the PCA biplot of gut microbiota (Figure S4B in the Supplementary Material), implying that the dietary fiber in brown beans played the main role in the modulation of gut microbiota. 

Both bean diets led to higher abundance of Firmicutes and a concurrent decrease in F/B ratio compared with the HF diet (Figure 5). A similar effect has been reported for another common bean, white kidney bean, in C57BL/6 mice [33]. A higher F/B ratio has been found in obese mice and humans compared with their lean counterparts [9,10], but in the present study, we did not observe lower fat accumulation in the bean groups compared with the HF group (Figure S2 in the Supplementary Material). This discrepancy may be due to the use of *Apoe*^−/−^ mice with a genetic defect in lipid metabolism. Another interesting observation was that there was no significant difference between the HF and two bean groups in relative abundance of the genus *Akkermansia*, which has been suggested to play an important role in alleviating metabolic disorders and atherosclerosis [34,35]. Enhanced abundance of *Akkermansia* has previously been reported in the gut microbiota of mice fed white kidney beans [33]. The discrepancy between studies might be explained by proliferation of the phylum Bacteroidetes in mice fed the brown bean diets. It is also worth noting that high abundance of *Akkermansia* (17%) was found in mice fed the HF diet, as reported previously by Jakobsdottir et al. [36]. Furthermore, we found that both bean groups had a higher abundance of unclassified *S24-7*, *Prevotella*, *Bifidobacterium*, and unclassified *Clostridiales*, which was in line with findings that supplementation with navy and black beans can stimulate the growth of fiber-fermenting bacteria, including *Prevotella, S24-7*, and *Ruminococcus*, in C57BL/6 mice [4]. Both bean diets reversed the decline in *Bifidobacterium,* a well-acknowledged beneficial microbe, induced by the HF diet (Figure 6B). Similarly, diet supplementation with whole kidney bean has been found to increase the relative abundance of *Bifidobacterium* in rats [32]. The increase is probably due to ability of *Bifidobacterium* to digest dietary fiber and other complex carbohydrates [37]. Furthermore, the HF diet led to an increase in *Lactobacillus* compared with the LF diet (Figure 6B). Although *Lactobacillus* is usually considered beneficial, some studies suggest that a high fat diet could promote the growth of some specific species of “bad” *Lactobacillus* associated with obesity [38,39]. Our data indicated that the brown bean interventions could reverse the increased abundance of *Lactobacillus* driven by the HF diet. 

### 4.2. Effects of Bean Diets on Microbiota-Dependent Metabolites

Higher pools of both total and individual SCFAs in the two bean groups were observed compared with the HF group (Figure 2). This was expected, because the brown bean diets were rich in fermentable carbohydrates compared with the control diets containing cellulose, which is highly resistant to microbial fermentation. Brown bean fiber is mainly composed of arabinose, glucose, uronic acids, and xylose (Figure S1 in the Supplementary Material), which is in line with the previous reports on the predominant neutral sugars of bean fibers [29,40]. The result also implies that cellulose and arabinoxylans were the main constituents. Although knowledge is still limited regarding the bacteria that use arabinoxylans, in an in vitro human intestinal bacterial culture study, the high formation of propionic acid was associated with arabinoxylans [41]. This could be the reason for the high cecal pools of propionic acid in mice fed brown beans (Figure 2). With regard to butyric acid, which is of special interest due to the potent effects in maintaining gut health and intestinal barrier function [42,43], the Wbean diet resulted in higher cecal pool in the mice and showed a tendency (*p* = 0.1) to increase the proportion of butyric acid compared with the HF diet (Figure 2 and Figure S3 in the Supplementary Material). That might be attributable to the presence of resistant starch and oligosaccharides in the Wbean fraction. Unfortunately, most of the resistant starch and oligosaccharides were lost during the preparation of the fiber fraction [44]. According to the literature data, the content of oligosaccharides and resistant starch in cooked brown bean is 3.2% and 7.6% dwt, respectively [21]. It has been reported that resistant starch and oligosaccharides can increase butyric acid formation in human subjects [45,46,47]. Besides, both bean diets resulted in higher cecal pools of minor acids compared with the HF diet (Figure 2). Minor acids, including branched SCFAs, are associated with fermentation of the indigestible proteins in beans [48]. 

Plasma TMAO concentrations were higher in the two bean groups than in the HF group (Figure 3). This is an interesting finding, because a high plasma concentration of TMAO is often associated with a risk of atherosclerosis [49]. However, the two bean groups with higher plasma concentrations showed a tendency (*p* = 0.1 and 0.07) to decrease the atherosclerotic plaque amount. This might be due to the use of *Apoe*^−/−^ mice, because in several other studies, *Apoe*^−/−^ mice with higher plasma TMAO concentrations have been found not to develop higher atherosclerotic plaque amount [50,51,52]. It is also worth mentioning that we used female mice, which are reported to have high expression levels of TMA-converting enzymes, resulting in higher basic plasma TMAO concentrations [53]. Furthermore, we observed significant differences in plasma choline between the two bean groups. The differences in plasma methylamines might also be partly attributable to higher dietary intake of feed in the bean groups compared with the control groups. Apart from choline bitartrate (0.25%), which was added to all diets, all but the LF diet contained lard, with 50 mg/100 g choline [54], and pulses contain up to 119 mg/100 g fresh weight of both choline and betaine [55]. Besides, mammals can endogenously synthesize methylamines [56]. Notably, compared with the HF diet, the Bfiber diet resulted in lower plasma creatinine concentrations and the Wbean diet showed a lowering tendency (Figure 3). As high plasma creatinine concentration is considered a predictor of renal disease [57], the results hint at potential protective effects of the bean fiber fraction against kidney disease. 

The differences in gut microbiota between the groups could also have resulted from differences in plasma concentrations of methylamines, since gut microbiota are responsible for converting the precursors to TMA. However, little is known about the bacteria that convert specific precursors. The bacterial taxa correlated to TMAO production identified in the present study belong to Deferribacteres, Firmicutes, and Proteobacteria, which is in line with previous findings [19,58]. Our data may confirm a relationship between microbiota and TMAO formation in the *Apoe*^−/−^ mouse model. 

### 4.3. Effects of Bean Diets on Plasma Lipid Profile and Atherosclerosis Plaque Amount

Neither of the bean diets affected plasma lipid profile compared with the HF diet (Figure 1). This was unexpected, since many animal studies suggest a hypolipidemic effect of bean foods [32,59,60,61]. Diet supplementation with dietary fiber from white kidney bean was reported to reduce total cholesterol in the serum of diabetic rats [59]. Yeap et al. [60] found that feeding diets containing fermented mung beans to hypercholesterolemic mice significantly reduced their serum levels of triglycerides, cholesterol, and LDL cholesterol. Similar cholesterol-lowering effects of pinto beans have been observed using a hamster model [61]. We observed that mice fed the bean diets had higher diet consumption and body weight gain (Figure S2 in the Supplementary Material), which was also inconsistent with previous findings of the weight-lowering effects of another common bean (white kidney bean) in obese mice [33]. The discrepancy between studies in the effects of the two bean types on body weight might be attributable to differences in the bean species, the mouse model (*Apoe*^−/−^ mice in our study vs. C57BL/6 mice in the literature), and the composition of the HF diet (37% fat (lard) in our study vs. 60% (palm oil) in the literature). The *Apoe*^−/−^ mice were characterized by dyslipidemia, which can easily result in hyperglycemia and excess diet intake [62].

The LF diet resulted in lower total and LDL cholesterol in plasma and lower atherosclerotic plaque amount compared with the HF diet (Figure 1), which is in line with Matziouridou et al. [63]. We also observed that the Wbean and Bfiber diets showed a tendency to lower atherosclerotic plaque amount compared with the HF diet. High blood cholesterol is a major risk factor for cardiovascular disease, and a reduced blood level of total cholesterol decreases the risk [64]. However, the bean diets did not affect plasma cholesterol levels (Figure 1). Thus, other factors might have contributed to the plaque-decreasing trend. One suggestion is that the bean diets reduced systematic inflammation, as a cross-sectional human study showed an inverse association between legume consumption and systematic inflammation [65]. It would be interesting to study the plasma levels of inflammatory markers, such as TNFα, and the colonic mRNA expression levels of inflammatory cytokines, such as IL-6 [65,66]. However, it is a limitation of our study that inflammatory markers were not analyzed, but could be a continuation for another study. The SCFAs produced by gut microbiota have also been proven to lower systematic inflammation in previous studies [49]. Interestingly, the bean diets enhanced the formation of cecal SCFAs (Figure 2), and the metabolic parameters regarding SCFAs were positively correlated with the mice in bean groups in PCA (Figure S4 in the Supplementary Material). Previous studies have suggested that the gut microbiota can be a factor in the progression of atherosclerosis [67]. In the present study, we identified two bacterial genera, *Streptococcus* and *Ruminococcaceae*, that were correlated with atherosclerotic plaque amount, which is in line with a previous suggestion that *Streptococcus* species can accelerate the increase in atherosclerotic plaques in *Apoe*^−/−^ mice [68].

In summary, both the Wbean and Bfiber diets resulted in a tendency for lower atherosclerotic plaque amount, but did not positively affect body weight and plasma lipid profile compared with the HF diet. The Wbean diet led to a higher alpha diversity of gut microbiota compared with the HF control. Both bean diets resulted in higher relative abundance of Actinobacteria and Bacteroidetes, and lower F/B ratio. At the genus level, both bean diets showed similar effects on the stimulation of the growth of bacterial genera, such as unclassified *S24-7*, *Prevotella*, *Bifidobacterium*, and unclassified *Clostridiales*, and led to lower relative abundance of *Lactobacillus.* The Bfiber fraction also resulted in lower abundance of *Oscillospira*. A higher formation of all cecal SCFAs, a higher proportion of cecal propionic acid, and a lower proportion of cecal acetic acid were observed in mice on both bean diets. The bean diets also resulted in higher plasma TMAO concentrations compared with the HF diet, while the Bfiber diet led to lower plasma creatinine concentrations and the Wbean diet showed a lowering tendency. Brown bean is a traditional and important type of pulse consumed in Sweden. The present study reveals the in vivo effects of both the whole bean and bean-derived fiber fraction on the plasma lipids, atherosclerotic plaque amount, gut microbiota, and cecal SCFAs. Bean-derived dietary fiber is often the main component of bean byproducts. As the fiber fraction contributes considerably to the nutritional benefits of the whole bean, the value and utility of fiber-rich bean byproducts should not be neglected.

## Data Availability

The data presented in this study are available in the article, Supplementary Material, or upon request to the authors.

## Supplementary Materials

The following supporting information can be downloaded at: https://www.mdpi.com/article/10.3390/nu14050937/s1, Table S1: Composition of the experimental diets; Table S2: Nutritional content and main flavonoids in the cooked whole brown bean (Wbean) and the isolated dietary fiber fraction (Bfiber); Figure S1: Monomeric composition (%) of total dietary fiber polysaccharides in cooked brown bean containing 27 g/100 g fiber on a dry weight basis; Figure S2: (A) Body weight gain, (B) cumulative diet consumption, (C) ratios of liver to body weight, (D) ratio of cecum to body weight, and (E) ratio of fat pads to body weight in mice fed a low fat (LF), high fat (HF), whole brown bean (Wbean), or bean fiber (Bfiber) diet for 10.5 weeks; Figure S3: Cecal proportions (%) of (A) acetic acid, (B) propionic acid, (C) butyric acid, and (D) minor acids in *Apoe*^−/−^ mice fed a control diet (low fat (LF) or high fat (HF)) or a bean diet (whole brown bean (Wbean), bean fiber (Bfiber)) in an HF setting for 10.5 weeks; Figure S4: Principal component analysis (PCA) biplot based on (A) organ and body weight and biomarkers and (B) bacteria obtained from mice fed a low fat (LF), high fat (HF), whole brown bean (Wbean), or bean fiber (Bfiber) diet for 10.5 weeks.
