# Supplementary material for: Effects of Whole Brown Bean and Its Isolated Fiber Fraction on Plasma Lipid Profile, Atherosclerosis, Gut Microbiota, and Microbiota-Dependent Metabolites in Apoe−/− Mice
Source: Nutrients. 2022 Feb 22;14(5):937. doi: 10.3390/nu14050937 (PMC8912725; doi:10.3390/nu14050937)
Supplement: Supplementary file 1 [file nutrients-14-00937-s001.zip › nutrients-1583933-supplementary.pdf]

J.L., M.E.H., C.M.W., M.B., S.B., M.N., F.H. (2022). Effects of whole brown bean and its isolated fiber fraction on plasma lipid profile, atherosclerosis, gut microbiota, and microbiota-dependent metabolites in *Apoe*$^{-/-}$ mice

Supplementary Material

Table S1. Composition of the experimental diets[1]: LF, low fat; HF, high fat; Wbean, whole bean; Bfiber, bean fiber

	LF[2]	HF[2]	Wbean	Bfiber
**Energy (kcal)**				
Soybean oil	450	450	450	450
Lard	0	1260	1260	1260
Casein	622.8	622.8	622.8	622.8
Sucrose	200	200	200	200
Vitamin mix	40	40	40	40
L-cystine	7.2	7.2	7.2	7.2
Maltodextrin 10	1000	0	0	0
Whole brown bean[3,4]	0	0	644.4	0
Brown bean fiber[3,5]	0	0	0	171.4
Corn starch	1768.5	2179.6	1535.2	2008.4
**Energy per gram (kcal/g)**				
Kcal/g, diet	3.8	4.4	4.4	4.4
**Energy composition (kcal%)**				
Protein	14	12	15	14
Carbohydrate	75	52	47	49
Fat	11	37	37	36

[1] Produced by Research Diets, Inc., NJ, USA. The background diet is a modified AIN-93M mature rodent diet (Reeves, 1997)
[2] Dietary fiber from cellulose, 60 g/kg dwt.
[3] The nutritional content in cooked whole bean and the fiber fraction is presented in Table S2.
[4] Resulting in 60 g/kg dwt dietary fiber in the final Wbean diet.
[5] Resulting in 60 g/kg dwt dietary fiber in the final Bfiber diet.

J.L., M.E.H., C.M.W., M.B., S.B., M.N., F.H. (2022). Effects of whole brown bean and its isolated fiber fraction on plasma lipid profile, atherosclerosis, gut microbiota, and microbiota-dependent metabolites in *Apoe*$^{-/-}$ mice

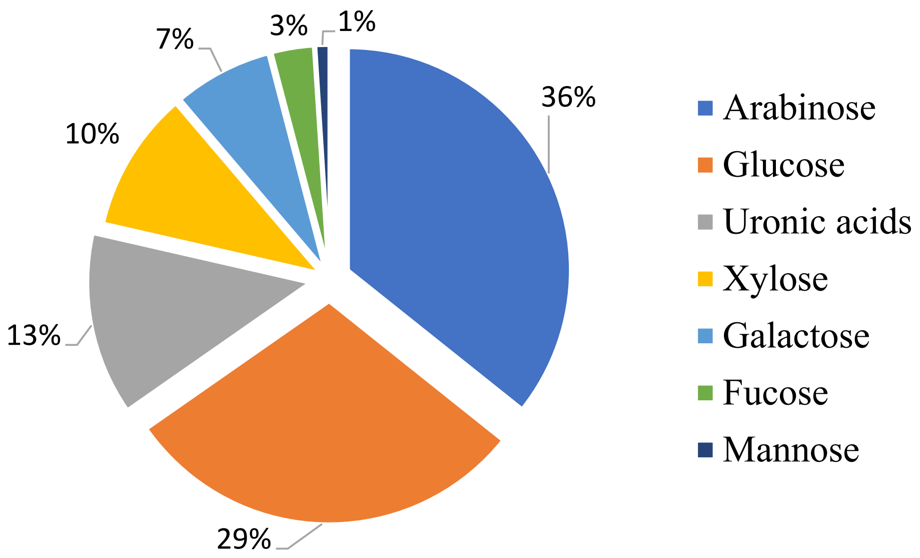

Figure S1. Monomeric composition (%) of total dietary fiber polysaccharides in cooked brown bean containing 27 g/100 g fiber on a dry weight basis.

J.L., M.E.H., C.M.W., M.B., S.B., M.N., F.H. (2022). Effects of whole brown bean and its isolated fiber fraction on plasma lipid profile, atherosclerosis, gut microbiota, and microbiota-dependent metabolites in *Apoe*$^{-/-}$ mice

Table S2. Nutritional content and main flavonoids in the cooked whole brown bean (Wbean) and the isolated dietary fiber fraction (Bfiber)

	**Wbean**	**Bfiber**
**Basic nutrients [1] (g/100 g, dwt)**		
Carbohydrates [2]	49	16
Protein	19	24
Fat	2	0
Ash	3	4
Dietary fiber [3]	27	56
**Polyphenols [4] (µg/g, dwt)**		
(+)-Catechin	42	n.d. [5]
Kaempferol-3-*O*-galactoside	329	n.d.
Quercetin-3-*O*-glucoside	12	n.d.
Kaempferol-3,6-malonyl-glucoside	21	n.d.
4-Hydroxybenzoic acid	16	n.d.
Caffeic acid	4	n.d.
p-Coumaric acid	5	n.d.
Ferulic acid	61	n.d.
Sinapic acid	23	n.d.
TOTAL	513	n.d.

[1] Content of nutrients (total dietary fiber (soluble and insoluble fiber), total protein, total fat, and ash) in the cooked whole bean and the fiber fraction were analyzed using corresponding AOAC methods (AOAC 985.29, 992.15, 989.05, and 923.03). Total carbohydrate was calculated by difference i.e., the total amount (100) minus other nutrients.

[2] Resistant starch and oligosaccharides were also included.

[3] Monomeric composition of dietary fiber is shown in Fig. S1.

[4] Quantification based on the method by Liu *et al.* (2021).

[5] n.d., not detected.

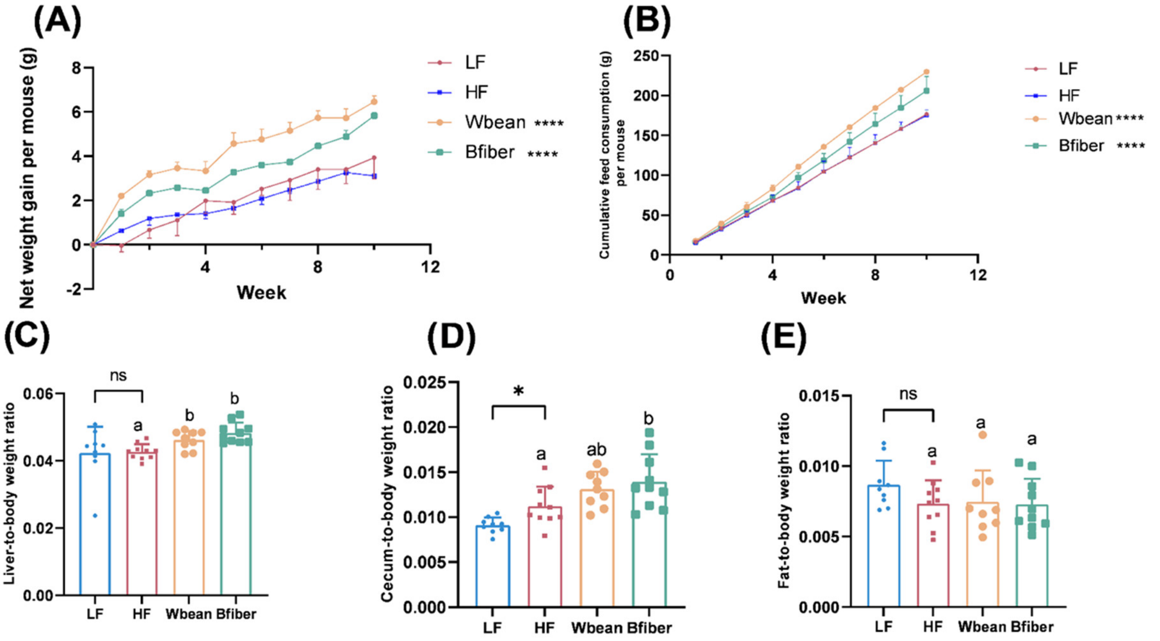

Figure S2. (A) Body weight gain, (B) cumulative diet consumption, (C) ratios of liver to body weight, (D) ratio of cecum to body weight, and (E) ratio of fat pads to body weight in mice fed a low fat (LF), high fat (HF), whole brown bean (Wbean), or bean fiber (Bfiber) diet for 10.5 weeks. Values in the LF and HF controls were compared using Student's *t*-test. Significant differences are marked in the LF column. Values in the high fat groups, i.e., HF, Wbean, and Bfiber, were compared using one-way ANOVA (Welch's ANOVA test for data with unequal SDs, or Kruskal-Wallis test for nonparametric data) followed by post hoc tests. Different letters on the columns indicate significant difference (p<0.05). ns, not significant. * p<0.05, **** p<0.0001.

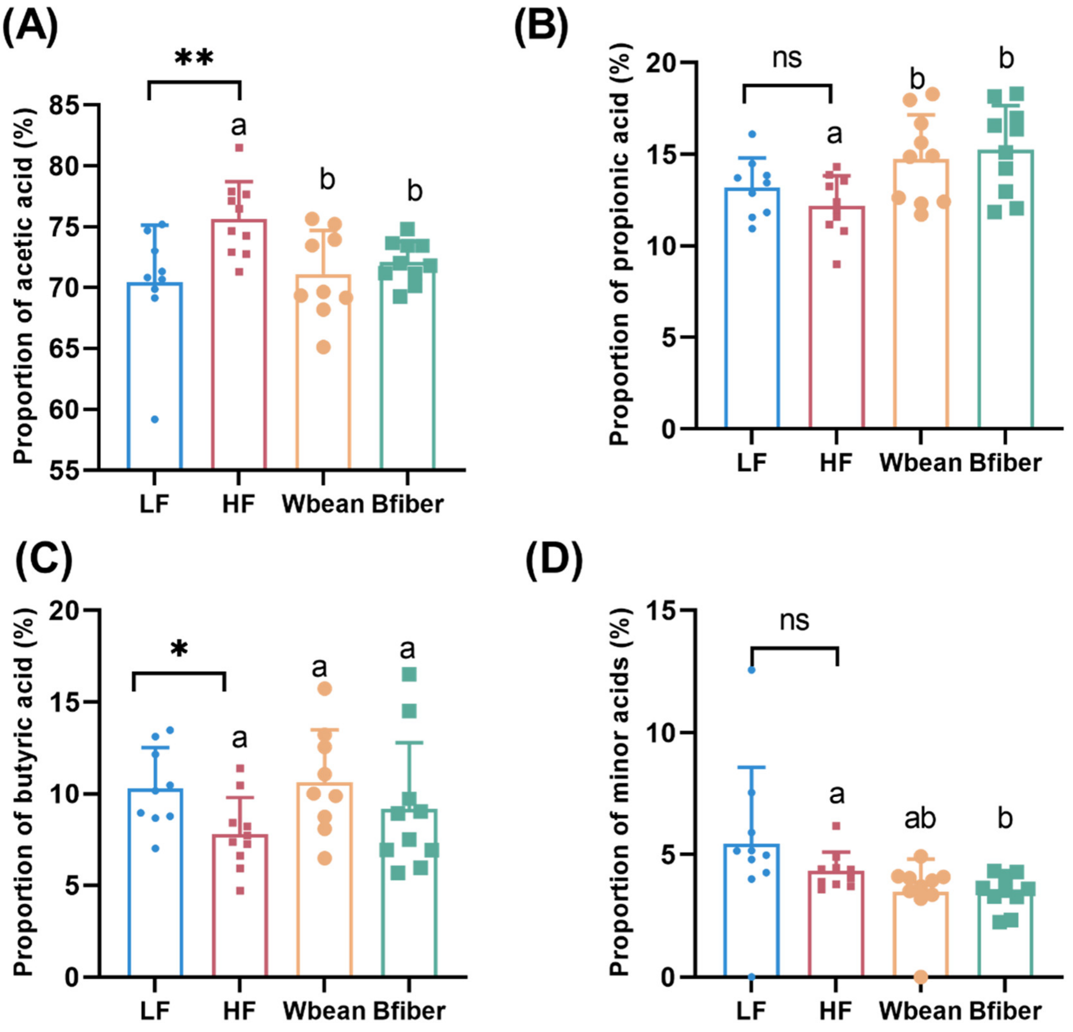

Figure S3. Cecal proportions (%) of (A) acetic acid, (B) propionic acid, (C) butyric acid, and (D) minor acids in *Apoe*$^{-/-}$ mice fed a control diet (low fat (LF) or high fat (HF)) or a bean diet (whole brown bean (Wbean), bean fiber (Bfiber)) in a HF setting for 10.5 weeks. Minor acids include isobutyric acid, valeric acid, isovaleric acid, caproic acid, and heptanoic acid. Values in the LF and HF controls were compared using Student's *t*-test. Values in the high fat groups, i.e., HF, Wbean, and Bfiber, were compared using one-way ANOVA followed by Tukey's multiple comparison test. Different lowercase letters within panels indicate significant differences ($p<0.05$). ns, not significant; * $p<0.05$, ** $p<0.01$.

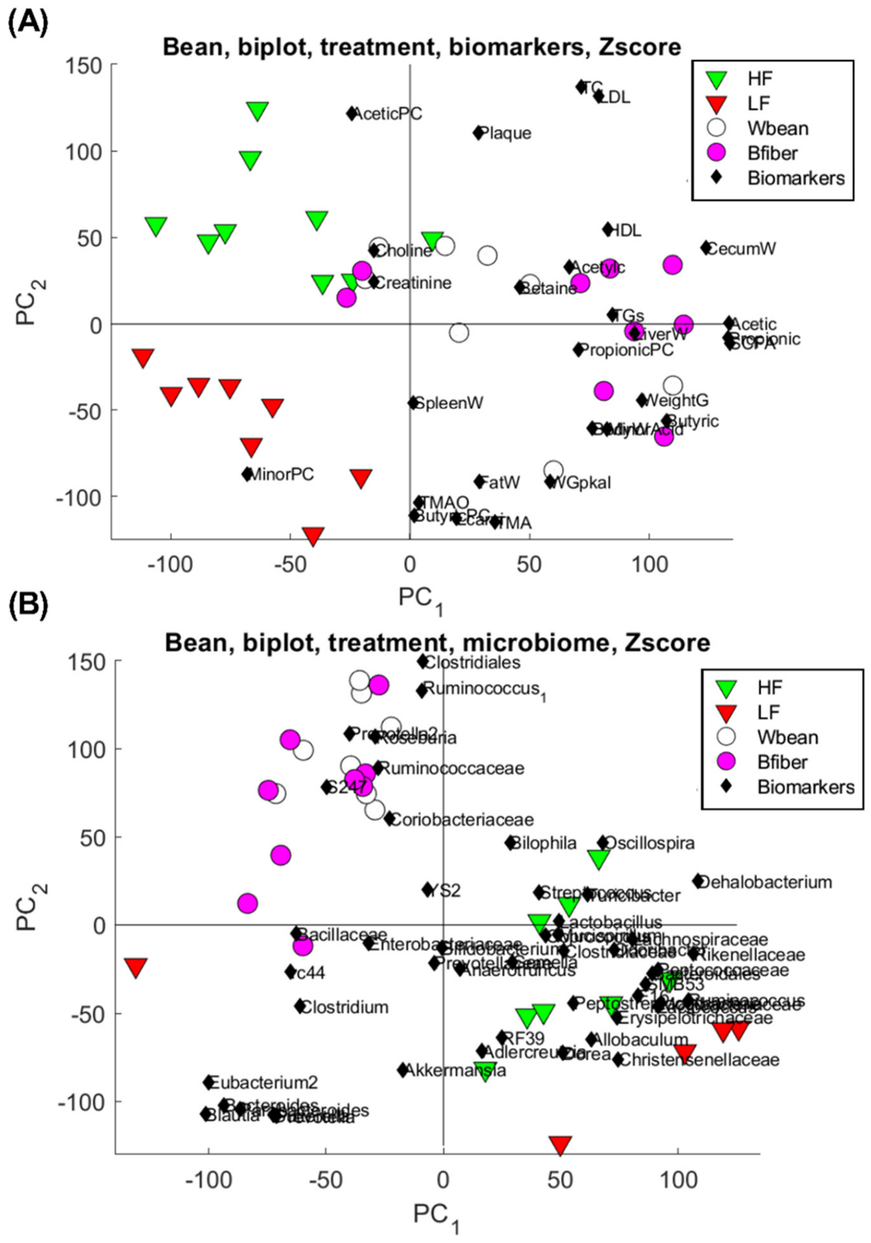

Figure S4. Principal component analysis (PCA) biplot based on (A) organ and body weight and biomarkers and (B) bacteria obtained from mice fed a low fat (LF), high fat (HF), whole brown bean (Wbean), or bean fiber (Bfiber) diet for 10.5 weeks. Symbols with different colors and shapes indicate mice in different groups. BodyW, body weight; LiverW, liver weight; CecumW, cecum tissue and content weight; FatW, fat pad weight; SpleenW, spleen weight; WeightG, weight gain; WGpkal, weight gain per kcal feed intake; TGs, triglycerides in plasma; TC, total cholesterol in plasma; HDL, high-density lipoprotein cholesterol in plasma; LDL, low-density lipoprotein cholesterol in plasma; SCFA, total amount of short-chain fatty acids in cecum; Acetic, total amount of acetic acid in cecum; Propionic, total amount of propionic acid in cecum; Butyric, total amount of butyric acid in cecum; MinorAcid, total amount of minor SCFAs in cecum; AceticPC, proportion of acetic acid to total SCFAs analyzed; PropionicPC, proportion of propionic acid to total SCFAs analyzed; ButyricPC, proportion of butyric acid to total SCFAs analyzed; MinorPC, proportion of minor SCFAs to total SCFAs analyzed; TMAO, plasma trimethylamine *N*-oxide concentration; Choline, plasma choline concentration; Creatinine, plasma creatinine concentration; Betaine, plasma betaine concentration; Acetylc, plasma acetyl-carnitine concentration; Lcarni, plasma L-carnitine concentration; TMA, plasma trimethylamine concentration; Plaque, atherosclerotic plaque amount.
